# Nutritional Supplementation of Naturally Occurring Vitamin D to Improve Hemorrhagic Stroke Outcomes

**DOI:** 10.3389/fneur.2021.670245

**Published:** 2021-07-30

**Authors:** Rani Ashouri, Madison Fangman, Jordan Brielmaier, Zoe A. Fields, Natalie Campo, Sylvain Doré

**Affiliations:** ^1^Department of Anesthesiology, University of Florida College of Medicine, University of Florida, Gainesville, FL, United States; ^2^Departments of Psychiatry, Pharmaceutics, Psychology, and Neuroscience, Center for Translational Research in Neurodegenerative Disease, McKnight Brain Institute, University of Florida, Gainesville, FL, United States

**Keywords:** herbal medicine, pathology, ischemia, bioavailabiiity, 25(OH)D deficiency

## Abstract

Vitamin D deficiency, if left untreated, is associated with bone disorders, cardiovascular damage, and an increased risk of ischemic stroke. While there are various nutritional options for the natural intake of vitamin D, we hope to elucidate the potential mechanisms dietary vitamin D may play in hemorrhagic stroke pathology. This scoping review outlines findings from studies relevant to the biochemical activity of vitamin D, the impact of vitamin D deficiency on hemorrhagic stroke outcomes, and the potential benefit of nutritional vitamin D on hemorrhagic stroke outcomes. Here, we analyze the relevant factors that can lead to vitamin D deficiency, and subsequently, a higher risk of hemorrhagic stroke incidence with worsened subsequent outcomes. The neuroprotective mechanisms through which vitamin D works to attenuate hemorrhagic stroke onset and post-stroke outcomes have not yet been thoroughly examined. However, researchers have proposed several potential protective mechanisms, including reduction of blood brain barrier disturbance by inhibiting the production of reactive oxygen species, mitigation of inflammation through a reduction of levels of proinflammatory cytokines, and prevention of cerebral vasospasm and delayed cerebral ischemia following subarachnoid hemorrhage and intracerebral hemorrhage. While more research is needed and there are limitations to vitamin D supplementation, vitamin D as a whole may play a significant role in the dynamics of hemorrhagic stroke. Further research should focus on expanding our understanding of the neuroprotective capacity and mechanisms of vitamin D, as well as how vitamin D supplementation could serve as an effective course of treatment of hemorrhagic strokes.

## Introduction

Vitamin D is a fat-soluble secosteroid hormone primarily responsible for regulating calcium and phosphorus levels as well as several other physiological functions (1). Cholecalciferol is produced in the skin in the presence of ultraviolet light, which is then hydroxylated into 25-hydroxyvitamin D (25(OH)D), calcifediol, in the liver and then again in the kidneys into 1,25-dihydroxy-vitamin D (1,25(OH)_2_D) or calcitriol, which is the active metabolite, transported systemically via vitamin D binding protein, capable of entering the cell and binding to an intracellular vitamin D receptor (VDR) (2). Calcitriol-bound VDR functions in the regulation of a variety of genes (3). As a transcription factor, VDRs control growth, differentiation, and functional activity of various cell types. These changes occur in the immune system, skin, pancreas, and various bone cells, primarily regulating mineral metabolism. VDRs and their respective target genes are thus most common in these cell types (3).

### Vitamin D Deficiency

Vitamin D deficiency and insufficiency has become a global health issue. Roughly one billion people globally suffer from vitamin D deficiency, and almost 50% of the world's population is vitamin D insufficient (4). Currently, there is no universally accepted definition of vitamin D deficiency; however, it is generally classified when levels of vitamin D are ≤ 12 ng/mL (30 nmol/L) (5–7). Insufficiency is categorized when vitamin D levels are below the clinical standard of 30 ng/mL (75 nmol/L) and above deficient levels (5–7). Risk factors for both vitamin D deficiency and insufficiency include older age, darker complexion, obesity, and limited sun exposure (8). While low levels of vitamin D have been an issue of a public health concern due to the increased risk of bone pathologies, such as bone demineralization or congenital bone abnormalities, more recently, researchers have shifted their focus to the systemic implications of vitamin D deficiency, such as hypertension, obesity, diabetes, cardiovascular disease, and cancer (9, 10). The preeminent source of vitamin D is sun exposure, which catalyzes the conversion of cholesterol into vitamin D. For sufficient vitamin D synthesis, it is recommended that outdoor sun exposure should last anywhere between 5 and 30 min, particularly between the hours of 10 am and 3 pm (depending on the season and the latitude), daily or a minimum of twice a week. Sunlight exposure to the face, arms, legs, and hands is most effective without sunscreen; however, exposure with sunscreen is still sufficient (11, 12). The efficacy of vitamin D synthesis by sun exposure varies based on the season, time of day, cloud coverage, geographical location, skin melanin content, age, and sunscreen use (13). When considering the numerous variables that may contribute to vitamin D deficiency, we turn toward the possibility of including dietary vitamin D as an option to establish regimented natural supplementation.

### Dietary Vitamin D

One way to reduce the prevalence of vitamin D deficiency may be incorporating natural sources of vitamin D into the diets and lifestyles of the general population. Dairy products without fortification, such as cheese, contain vitamin D and its metabolite, 25(OH)D. Additional sources that naturally produce vitamin D include fatty fish such as salmon (570 IU/14.2 μg per serving), cod liver oil (1,360 IU/34 μg per serving), trout (645 IU/16.2 μg per serving), tuna (40 IU/1 μg per serving), and sardines (46 IU/1.1 μg per serving). Eggs (44 IU/1.1 μg per egg) and mushrooms (366 IU/9.2 μg per serving) also naturally contain vitamin D per the Institute of Medicine (US) Committee to Review Dietary Reference Intakes for Vitamin D and Calcium. Countries around the world have different vitamin D intake guidelines due to an incomplete understanding of the biological and clinical implications of vitamin D. For example, to combat the high rates of vitamin D deficiency, the Endocrine Society recommends daily dietary vitamin D supplements of 1,500–2,000 IU every day for adults and at least 1,000 IU for children and adolescents (14). On the other hand, the United Kingdom government recommends daily supplementation of 400 IU for all citizens over the age of three, which further emphasizes a need for universal guidelines regarding vitamin D intake and maintenance (15). Dietary supplements containing vitamins D2 and D3 are both readily available in consumer markets. The distinction between these two options of supplements is significant, and it is important for clinicians, consumers, and patients to be aware of the differences before their incorporation into therapeutic regimens (see Table 1). Both ergocalciferol (vitamin D2) and cholecalciferol (vitamin D3) are effective at increasing serum 25(OH)D levels in the body; however, evidence shows that vitamin D3 raises these levels to a greater extent and can maintain higher levels of serum 25(OH)D for longer periods of time than vitamin D2, suggesting that vitamin D3 is a preferred choice for supplementation (16). Two randomized controlled trials on raising vitamin D levels from insufficient and deficient levels were conducted. One study reported that patients with an initial 25(OH)D level of 8 to <16 ng/mL, 16 to 24 ng/mL, and 24 to 32 ngL/mL required 2,200, 1,800, and 1,160 IU of cholecalciferol, respectfully, to reach 30 ng/mL (17). A more recent prospective cohort study by van Groningen et al. analyzed the daily IU of cholecalciferol required to raise serum 25(OH)D levels to normal levels in a sample of 208 patients. It was found that patients with initial 25(OH)D serum levels of 8 to <16 ng/mL, 16 to 24 ng/mL, and 24 to 32 ng/mL required 1,875 to 2,750 IU, 750 to 1,875 IU, and up to 750 IU, respectively, depending on the body weight (18, 19). Thus, via the inclusion of a few servings of the aforementioned vitamin D-rich foods, the hypothetical equivalent supplementary vitamin D dosage comparable to synthetic production could be reached naturally and nutritionally.

**Table 1:** Summary of ergocalciferol supplementation compared to cholecalciferol supplementation.

**Vitamin D_**2**_ (Ergocalciferol)**	**Vitamin D_**3**_ (Cholecalciferol)**
Found most commonly in plants and fungi	Naturally produced in the skin and the oil of fur (Dark skin requires 4x sun exposure to acquire the same dose)
Synthetically derived supplement(An alternative for strict vegetarians or vegans)	Naturally derived supplement
Moderately increases serum vitamin D	Significantly increases serum vitamin D (Displays a 3–10-fold differential potency compared to vitamin D_2_)
Microsomal 25-hydroxylase does not act on vitamin D_2_	Substrate for both microsomal and mitochondrial 25-hydroxylases
Serum 25(OH)D response to vitamin D_2_ supplementation is less in the elderly than in young adults	Serum 25(OH)D response to vitamin D_3_ supplementation is the same in both groups
Several known cases of iatrogenic toxicity with vitamin D from vitamin D_2_ usage	Known cases toxicity from vitamin D_3_ have been accidents (Too much may increase risk of calcium deposits in blood vessels)
Less stable in dose preparations	More stable in dose preparations
Concentration increases rapidly declines over time after a 3 day period	Concentration levels maintain over time

*Vitamin D binding protein has lower affinity for vitamin D_2_ as compared to vitamin D_3_*.

### Bioavailability and Absorption

To combat vitamin D deficiency and improve patient outcomes following a hemorrhagic stroke, it is necessary to consider nutritional methods of obtaining sufficient amounts of vitamin D. Considering the availability of high vitamin D levels found in cod liver oil, trout, salmon, and mushrooms, a dietary focus on raising serum levels of vitamin D should be evaluated for its potential in providing neuroprotection against stroke outcomes.

There are a few factors that affect nutritional vitamin D absorption and bioavailability. Ergocalciferol is the form of vitamin D present in fortified foods and common dietary supplements, while cholecalciferol is the main dietary form. Although ergocalciferol is absorbed similarly to cholecalciferol, calcifediol displays more efficient absorption than both the previously mentioned compounds. The increased absorption rate of calcifediol can be accredited to its hydroxyl group, which increases the polarity of the compound and allows it to be a water-soluble molecule (20). Moreover, it is proposed that drugs aimed to diminish obesity, such as sucrose polyesters and tetrahydrolipstatin, have the potential to inhibit or decrease vitamin D absorption, although more research into this idea is needed (21).

The three types of synthetic supplementary vitamin D delivery systems currently include oil, microencapsulated, and micellized forms. One experiment utilized Wistar rats to investigate the supplemental bioavailability of vitamin D and prepared the animals to receive the three different forms of vitamin D supplements over a series of weeks using a SmartHit IV model. The control level of cholecalciferol in the rat serum was recorded at a concentration of 36.49 ± 4.12 to 40.5 ± 3.05 nmol/L. Meanwhile, in the microencapsulated and oil-based treatment groups, levels reached 143.35 ± 14.72 and 150.85 ± 35.77 nmol/L, respectively. Vitamin D was recorded at the highest concentration in the oil-based cholecalciferol group on day 7, where it reached a peak concentration of 198.93 ± 51.6 nmol/L (22). These results indicate that microencapsulated and oil supplements were most effective in administering vitamin D as the encapsulation allowed the vitamin D to travel further into the digestive system, and the oil allowed for quicker digestion after it was released.

The rate at which vitamin D is processed within the gastrointestinal tract is partly controlled by several factors that directly link to vital lipids, such as phospholipids and triglycerides (23, 24). Furthermore, the acidity of gastric juices is a determining factor in vitamin D distribution and supplement processing. Pepsin and trypsin have been shown to play a role in cleaving the vitamin D proteins in food, which allows for its release into the system. With this data in mind, considerations could be made for the oil content and delivery of dietary vitamin D to maximize natural nutritional intake.

### Vitamin D Pathologies and Hemorrhagic Stroke

Vitamin D toxicity, also known as hypervitaminosis D, can occur as a result of excessive consumption of large doses of vitamin D supplements over an extended period of time, although it cannot occur from dietary intake or sun exposure alone. Hypervitaminosis D can cause hypercalcemia, a buildup of calcium in the blood, and can lead to nausea and vomiting, frequent urination, and muscle weakness. Vitamin D toxicity can also lead to the progression of osteodynia and kidney problems (25). While disease pathology relating to vitamin D toxicity warrants scientific discussion, the considerable prevalence of vitamin D deficiency is of great concern and will be discussed in the rest of the review. Vitamin D deficiency has been shown to increase the risk of various bone pathologies as well as be an independent risk factor for stroke, suggesting that vitamin D supplementation may serve as a potential therapy for stroke patients (26–31). While existing research focuses on the dynamics of vitamin D relating to ischemic stroke, we hope to elucidate the relationship between dietary inclusion of vitamin D and, specifically, hemorrhagic stroke. Studies have shown that hemorrhagic stroke patients often suffer from vitamin D deficiency or insufficiency with median vitamin D levels on presentation ranging from 10.5 nmol/L (32) to 42 nmol/L (33), both of which fall short of the clinical standard for vitamin D sufficiency. This review aims to analyze if a causal relationship exists between vitamin D deficiency and hemorrhagic stroke outcomes and evaluate if vitamin D-rich diets could improve these outcomes following the incidence of intracerebral hemorrhage (ICH) and subarachnoid hemorrhage (SAH).

Hemorrhagic stroke is a subtype of stroke that is caused by the rupturing of a blood vessel, resulting in bleeding in the brain or meninges and can be further classified as ICH or SAH. Spontaneous ICH makes up about 15–20% of all strokes and affects roughly 120,000 individuals in the United States each year (34); however, this number is expected to grow with the aging population (35). Furthermore, 78–88% of ICH cases are primary accounts that originate from the spontaneous rupture of small arteries in the brain that were damaged by either hypertensive arteriolosclerosis or amyloid angiopathy (35, 36). Secondary cases are far less common and are directly related to preexisting conditions such as coagulopathy, brain tumors, aneurysms, vascular anomalies, or thrombolytic treatment of ischemic stroke (35, 36). In all ICH incidents, blood accumulates in the brain and compresses the tissue surrounding the ruptured artery. Secondary brain damage, including a cascade of damaging inflammatory and oxidative events as well as dysfunction of the blood brain barrier, is commonly induced by ICH (37). Notably, this damage has been shown to be limited by treatment with 1,25(OH)_2_D_3_ *in vitro* (38). The second type of hemorrhagic stroke is SAH, which occurs due to bleeding in the brain between the subarachnoid space and the skull. This bleeding leads to a stroke by increasing pressure on the brain, damaging brain cells, and irritating the brain lining while depriving the affected artery of blood. SAH may be caused by a ruptured aneurysm, arteriovenous malformation, or traumatic brain injury (TBI) (39). One primary concern in the treatment of SAH is the prevention of cerebral vasospasm, a complication of the blood vessels that vitamin D levels may play a role in preventing.

## Biochemistry and Mechanisms of Vitamin D Protection in ICH and SAH

**Figure 1:**
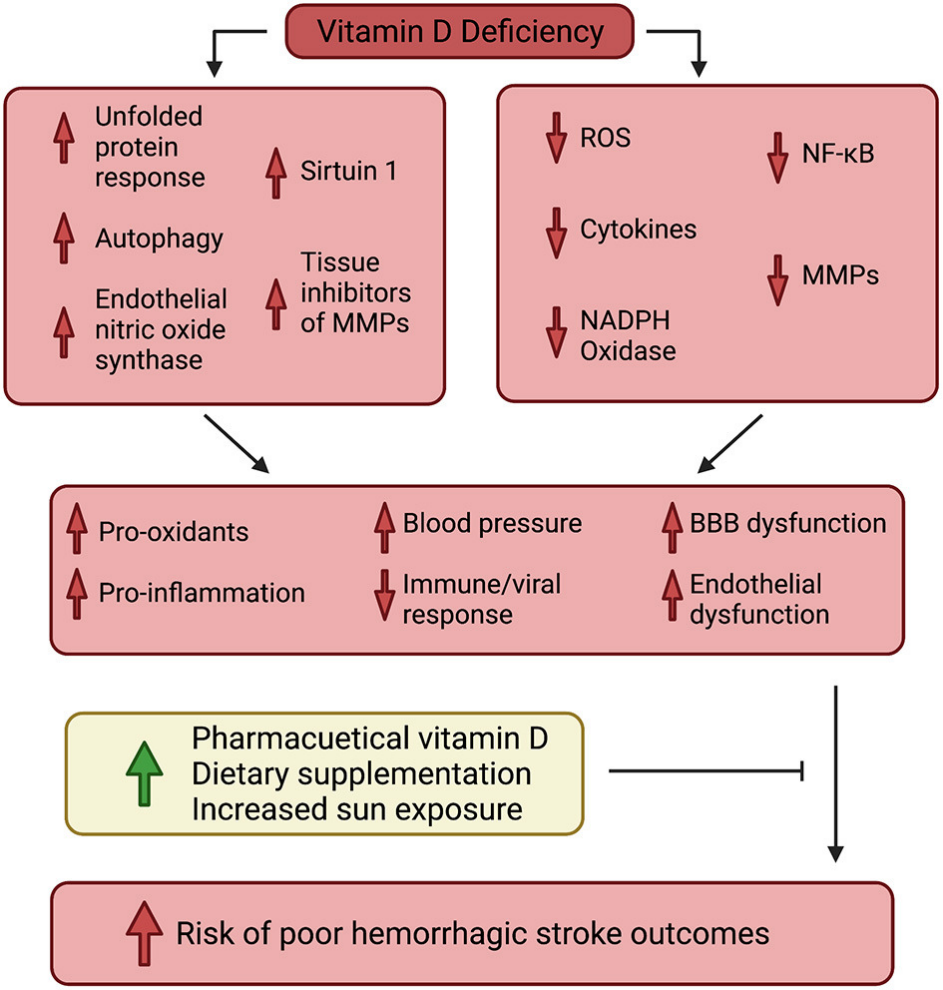
Visual schematic representing the dynamics of vitamin D's impact on hemorrhagic stroke patient health.

Vitamin D regulates a broad spectrum of physiological processes that contribute to cerebrovascular health and the potential for dysfunction. Activation of these processes, or inhibition of those active during vitamin D deficiency, could establish vascular inflammatory and oxidative conditions conducive to improved health outcomes following the incidence of hemorrhagic stroke. See Figure 1.

### Blood Brain Barrier

The blood brain barrier (BBB) is an important border between the circulatory system and the brain and functions to protect the neural tissue of the brain from contacting neurotoxic material or pathogens. The BBB works by regulating the exchange of substances from circulation to the brain. A defect or loss of integrity in the BBB can contribute to a variety of CNS diseases (40, 41). Epithelial tight junctions are critical features for maintaining the structural integrity of the BBB. There is a loss of BBB integrity during and shortly following a stroke episode due to increased tight junction vulnerability and BBB permeability. A main contributor to the BBB disturbance is the increased production of reactive oxygen species (ROS), which cause direct damage as well as upregulation of cell-disrupting proteins such as matrix metalloproteinases (MMPs) and nuclear factor kappa B (NF-κB). However, it has been shown that 1,25(OH)_2_D_3_/VDR interaction can protect against stroke-inflicted BBB disturbance via the inhibition of NF-κB activation and, therefore, downstream MMP-9 expression (38). NF-κB activation in neurons following a stroke leads to neuronal damage and cell death. Activation of NF-κB in astrocytes and endothelial cells in the BBB due to inflammation is associated with increased permeability of the BBB due to the disruption of tight junctions and opening of the BBB. NF-κB activation in pericytes causes MMP secretion, degrading the basement membrane and opening up the BBB to further damage (42). The VDR interacts with IκB kinase β (IKKβ) to block the activation of NF-κB. This VDR-IKKβ interaction is amplified by 1,25(OH)_2_D_3_ treatment (43). Therefore, via VDR activity, high levels of vitamin D are suggested to be directly neuroprotective via stabilization of the BBB post-stroke. Furthermore, vitamin D deficiency has been shown to contribute to downregulation of tissue inhibitors of MMPs, making vitamin D a critical candidate for protecting BBB integrity (44).

### Inflammation, ROS, and Vitamin D

There is an inflammatory response that occurs in the brain following the incidence of stroke. While acute inflammation may protect the brain by clearing deposited iron and encouraging neural plasticity, chronic inflammation displays damaging effects to both the brain and the BBB (45). ICH- and SAH-induced hemolysis leads to hemoglobin release, which subsequently releases heme and iron after degradation into the brain and meninges. These cytotoxins are a principal contributor to secondary brain injury following ICH. Free heme is a powerful neurotoxin that catalyzes oxidative reactions that induce damage to proteins, DNA, lipids, and the BBB, leading to irreversible impairment in the brain (46). Additionally, heme leads to cytokine upregulation and leukocyte activation (47). In excess, these molecules can impact signaling pathways in the brain and impair a variety of motor and sensory functions (48). Furthermore, hemoglobin and heme, when oxidized to methemoglobin and hemin, respectively, become high-affinity ligands for toll-like receptors, TLR-4 and TLR-2. TLR-4 signaling leads to activation of the inflammatory proteins NF-κB and TNFα along with production of ROS, further contributing to post-stroke neuroinflammation and BBB disturbance (47, 49). In ICH and SAH, accumulating arterial blood in the brain compresses the surrounding brain tissue and leads to necrosis, which signals the release of additional proinflammatory mediators (35). These mediators include proinflammatory cytokines such as interleukin 1 beta (IL-1β) and interleukin 6 (IL-6), which are released intracranially after a stroke and are responsible for local inflammation (50). The induction of IL-6 and chemokine CXCL-1 are dominant in the peripheral inflammatory response to stroke and can increase brain tissue injury as well as lead to BBB breakdown (51, 52). Once BBB permeability is increased, peripherally derived mononuclear phagocytes, T-lymphocytes, natural killer cells, and polymorphonuclear neutrophilic leukocytes, which produce cytokines, can all cross the BBB and further contribute to brain inflammation (53). Higher levels of proinflammatory cytokines, like IL-6, are also associated with worse neurological outcomes in patients post-stroke (54). Studies have reported that vitamin D deficiency elevates inflammatory protein levels, including IL-1β, TNFα, NF-κB, and IL-6, in the brain (55). It has also been shown that vitamin D deficiency leads to reduced modulatory autophagy and Sirtuin 1 and increased levels of NADPH oxidase. Overall, this molecular milieu leads to exacerbated oxidative conditions (44). Sufficient levels of vitamin D have been shown to attenuate this response, suggesting that it displays anti-inflammatory properties (56). Therefore, those with diets high in vitamin D could potentially avoid the deleterious effects of these proinflammatory molecules and resultant damage.

### Vitamin D and Osteopontin (OPN)

Osteopontin (OPN) is a neuroprotective glycoprotein that has been shown to attenuate the post-ICH inflammatory response and decrease brain injury induced by ICH while improving neurological function after a hemorrhagic stroke (57). While the interaction between OPN and levels of vitamin D is currently inconclusive, a few studies found that increasing levels of vitamin D through vitamin D receptor agonist (VDRA) therapy or *in vitro* treatment with 1,25(OH)_2_D_3_ upregulates OPN, while disrupting the VDR inhibits OPN transcription (58–60). Recombinant OPN (r-OPN) has been shown to suppress IL-1β-induced activation of the nuclear factor NF-κB and MMP-9 without affecting levels of IL-1β, which preclinically prevented BBB disruption following SAH (61). IL-1β is reported to activate three types of proteins that reduce the induction of MMP-9, including mitogen-activated protein kinase (MAPK), extracellular signal-regulated kinase (ERK), and c-Jun N-Terminal kinase (JNK), as well as NF-κB (62). The use of r-OPN has been shown to induce an endogenous MAPK inhibitor, MAPK phosphatase (MKP-1), in the spastic cerebral arteries and prevent cerebrovasospasm (63). Cerebral vasospasm is the delayed narrowing of large arteries located at the base of the brain. This narrowing is believed to be the most preventable secondary complication of SAH and a primary cause of morbidity and mortality (63, 64). Conversely, other studies have found a negative correlation between vitamin D and OPN expression, where vitamin D deficiency resulted in higher levels of OPN (65). Therefore, more research is necessary to clarify the definitive relationship between OPN and vitamin D levels and how this dynamic may impact ICH and SAH pathologies.

## Current Studies

It is postulated that vitamin D deficiency could lead to worsened stroke outcomes due to its anti-inflammatory properties and role in protecting the integrity of the BBB. This relationship has been studied in clinical and preclinical models, primarily centered around ischemic stroke. Few studies have been conducted regarding hemorrhagic strokes, although more research would prove beneficial to comprehensively elucidate the relationship between vitamin D deficiency and hemorrhagic stroke outcomes. While vitamin D deficiency has been shown to increase the severity of stroke symptoms, current research only shows some degree of association between vitamin D and stroke outcomes (29). There has been a direct correlation in patients with older age/malnutrition having a higher risk for severe clinical presentation of ICH (66). A meta-analysis of several stroke studies reported that higher dietary vitamin D levels were associated with a 24% reduction in cognitive impairment (28). Additional research suggests that 25(OH)D is inversely linked to chronic brain injury associated with small vessel disease, showing vitamin D deficiency was associated with lacunes, white matter damage, and microbleeds in the brain (95% CI: 0.04–0.95, 1.31–6.45, and 1.03–2.78, respectively) (67).

### Preclinical Studies With Vitamin D

Several preclinical trials using rats have addressed the anatomical and functional outcomes of vitamin D deficiency in stroke patients. Sayeed et al. showed that post-ischemia BBB breakdown was increased in Wistar rat models that were fed vitamin D deficient diets and displayed lower serum levels of vitamin D. BBB breakdown was measured by western blot analysis of IgG and MMP-9 levels present in 72-h post-ischemia infarct areas. The functionality of the tight junctions was also monitored through immunofluorescence and western blotting for claudin-5 and occludin expression to measure BBB permeability. Rats that were on a vitamin D deficient diet had increased BBB permeability according to IgG extravasation following middle cerebral artery occlusion (*p* < 0.01) and showed increased intensity of MMP-9 staining (*p* < 0.05) compared to rats given a diet containing sufficient levels of vitamin D. These results indicate that vitamin D deficiency would likely correlate with more severe complications after ischemic stroke (68). These findings are supported in another study that concluded mice fed vitamin D deficient diets showed a significant increase in infarct volume and decreased functionality post-ischemic stroke. Sprague-Dawley rat models were treated with normal control or vitamin D deficient diets and subjected to middle cerebral artery occlusion to model prolonged ischemia. Post-stroke functionality was then tested using sensorimotor and tape tests, finding that vitamin D deficient rats took longer to respond to tactile stimulus and remove the tape from their forearms (*p* < 0.05), inferring worse functional outcomes in vitamin D deficient stroke patients. Infarct volumes were also compared using brain slices and TTC-staining, revealing no significant difference in infarct volume at 3 days post-stroke. However, at 5 days post-stroke, there was a significant difference (*p* < 0.05) in infarct volume due to improvement of stroke tissue observed in the control rats that was not seen in the vitamin D deficient rats, revealing the possibility that vitamin D deficiency may result in worse stroke outcomes due to its potential inhibition of stroke tissue recovery (69). While the majority of preclinical studies have centered around vitamin D in terms of ischemic stroke outcomes, Enkhjargal *et al.*, studied SAH and vitamin D interactions to determine the effect of pretreating Sprague-Dawley rats with 30 ng/mL of calcitriol on stroke outcomes. It was found that the pretreated mice displayed better post-stroke neurological scores (*p* < 0.05), which suggests a therapeutic role of vitamin D in the treatment of hemorrhagic stroke (70).

### Vitamin D Supplementation Effect on Hemorrhagic Stroke Outcomes

A clinical trial by Narasimhan et al. found that vitamin D deficient ischemic stroke patients showed restored vitamin D levels and significant improvement in post-stroke outcome when treated with supplemental vitamin D. Sixty vitamin D deficient (vitamin D serum levels <20 ng/mL) or vitamin D insufficient (21–29 ng/mL) ischemic stroke patients participated in the study. Half of the participants received a single 6 lac IU injection of cholecalciferol upon arrival, while the other half were given no vitamin D supplements. Stroke outcomes for both groups were assessed by comparing stroke-severity scores upon arrival and 3 months post-treatment. The test group receiving the cholecalciferol treatment experienced greater improvement of stroke-severity scores than the untreated group. The test group experienced a 6.39 ± 4.56 score improvement, while the control group only showed a gain of 2.50 ± 2.20 (*p* < 0.001) (71). Contrastingly, a subsequent double-blind study of 100 participants was conducted to test the effects of cholecalciferol supplementation on post-ischemic and hemorrhagic stroke outcomes. This study found that there was no statistically significant difference in 8-week post-stroke outcomes assessing the Barthel Index score (*p* = 0.46), Brunnstrom stage, handgrip strength improvement (right *p* = 0.77; left *p* = 0.74), and calf circumference improvement (right *p* = 0.77; left *p* = 0.60) between the placebo group and the test group receiving 2,000 IU of oral cholecalciferol supplements daily (72). While variable results are expected, these contrasting outcomes may be a result of concluding the latter study too early. Due to the metabolism necessary to convert cholecalciferol to the active metabolite, calcitriol, more than 8 weeks may have been necessary to note efficacy in the vitamin D receiving group. Consideration for the significant time required for vitamin D bioactivity should be made in future research via longer trial times, such as the former study's three-month trial period.

### Vitamin D Deficiency Contribution to Post-stroke Anxiety, Depression, and Fatigue

Mental health can play a significant role in post-stroke outcomes and recovery. Low serum vitamin D levels and vitamin D deficiency have been linked to post-stroke anxiety, depression, and fatigue. It has been shown that patients who suffer from post-stroke anxiety compared to non-post stroke anxiety patients had significantly lower serum levels of vitamin D (*p* = 0.02). The post-stroke anxiety patients also exhibited poorer functional outcomes (modified Rankin scale *p* < 0.001; Barthel Index *p* < 0.001), worse cognitive function (mini-mental state examination *p* = 0.04), and more severe strokes (NIHSS *p* = 0.02) (73). Additionally, depression is observed in roughly one-third of all stroke patients (74). The patients with symptoms of post-stroke depression were found to have a mean vitamin D serum level of 8.3 ng/mL, while those not exhibiting depressive symptoms had a mean level of 15.6 ng/mL. Serum levels under 11.2 ng/mL were linked to a significant increase in post-stroke depression (95% CI 4.97–28.63; *p* < 0.001) as well as linked with worse NIHSS scores (*p* < 0.001) (74). Moreover, there is a high prevalence of vitamin D deficiency among patients who experience post-stroke fatigue, defined as persistent lack of energy and excessive physical tiredness following either ischemic or hemorrhagic stroke. Patients treated with loading doses of cholecalciferol for a month to replenish vitamin D levels self-reported significant improvement in post-stroke fatigue symptoms at follow-up appointments between 4 weeks and 6 months after the stroke incident (75). While these studies show a correlation between vitamin D deficiency and post-stroke mental illness development, they were almost exclusively conducted in ischemic stroke patients. Therefore, further research on the connections between vitamin D and mental health should be conducted in ICH and SAH patients to establish further the effects of vitamin D on overall wellness (73–75).

## Conclusion and Future Research

In conducting this review, it is evident that vitamin D deficiency has several implications that correlate with dynamic biochemistry inherent to hemorrhagic strokes. Vitamin D protects against BBB disturbance, suppresses cytokine production, as well as reduces cognitive impairment and inflammation. Although there are limitations to the studies, advancements within the research show a promising correlation. More research must be conducted, both preclinical and clinical in nature, to specifically evaluate the way nutritional vitamin D could impact the quality of life of patients after the incidence of hemorrhagic stroke. For clinical investigation, a retrospective study evaluating active vitamin D metabolite levels in patients and correlating findings with stroke outcomes should be designed. Additionally, it would be beneficial to design a prospective clinical study to screen stroke patients upon arrival to hospitals and measure their levels of vitamin D metabolite. Depending on each screening, vitamin D analogs should be given; accordingly, that would most rapidly transform to the active vitamin D metabolite. These findings would provide additional evidence of the intricacy of vitamin D in stroke pathology and subsequent outcomes.

## Study Design and Limitations

This scoping review was intended to encompass the existing research that is available regarding vitamin D, including its nutritional, supplementary, and endogenous forms and the impact it may have on outcomes following hemorrhagic stroke. Our search methodology consisted of the use of all standard short and long-form references to vitamin D, including: “Vitamin D, 25(OH)D” in a search pattern of (“Vitamin D” OR 25(OH)D OR calcitriol OR cholecalciferol OR ergocalciferol OR 25-hydroxyvitamin D OR 1,25-dihydroxyvitamin D) AND (“hemorrhagic stroke” OR ICH OR SAH OR “subarachnoid hemorrhage” OR “intracerebral hemorrhage”) in Pubmed, Embase, Google Scholar, OneSearch, and Web of Science. In addition to individual database searches, related literature present in the bibliographies of other analyzed papers was used to conduct a more comprehensive search. While this is a scoping review and not systematic in nature, the bias in literature selection was avoided, with each author doing independent searches and compiling studies into an aggregate list. After compilation of this aggregate list, no papers were excluded from the study. Clinical studies regarding vitamin D are generally subject to a degree of variability due to the roles that geographic location and temporal variations in sun exposure play in its production. While measuring patient serum vitamin D would ideally account for these deviations, ambulatory vitamin D levels could vary widely between clinic visits. Current research typically accounts for these variables, although the various dynamic factors that contribute to the biological production of vitamin D should be considered when reviewing related literature.

## Author Contributions

SD: conceptualization and funding acquisition. RA and SD: methodology and analysis. RA, MF, JB, ZF, and NC: writing—original draft preparation. RA, MF, JB, ZF, NC, and SD: writing—review and editing. All authors have read and agreed to the published version of the manuscript.

## Conflict of Interest

The authors declare that the research was conducted in the absence of any commercial or financial relationships that could be construed as a potential conflict of interest.

## Publisher's Note

All claims expressed in this article are solely those of the authors and do not necessarily represent those of their affiliated organizations, or those of the publisher, the editors and the reviewers. Any product that may be evaluated in this article, or claim that may be made by its manufacturer, is not guaranteed or endorsed by the publisher.

## Abbreviations

BBB: blood brain barrier
ICH: intracerebral hemorrhage
MAPK: mitogen-activated protein kinase
MMP: matrix metalloproteinase
NF-κB: nuclear factor kappa B
OPN: osteopontin
ROS: reactive oxygen species
SAH: subarachnoid hemorrhage
VDR: vitamin D receptor.
